# Mycotoxin Contamination Concerns of Herbs and Medicinal Plants

**DOI:** 10.3390/toxins12030182

**Published:** 2020-03-14

**Authors:** Iwona Ałtyn, Magdalena Twarużek

**Affiliations:** Department of Physiology and Toxicology, Faculty of Biological Sciences, Kazimierz Wielki University, Chodkiewicza 30, 85-064 Bydgoszcz, Poland; iwonaalt@ukw.edu.pl

**Keywords:** herbs, contamination, liquorice, chamomile, mint, ginseng, milk thistle, ginger, medicinal plants, mycotoxins

## Abstract

Plants and medicinal herbs that are available on the market do not always meet quality and safety standards. One particular concern is the risk of contamination with mycotoxins. Aflatoxins and ochratoxin A are the most frequently described mycotoxins in herbal products and have repeatedly been reported to occur at concentrations which exceed regulatory levels set by the European Union (EU). Possible solutions include enforcing existing limits, and for the new materials, establishing tighter limits and mandate the growth of medicinal plants in EU member countries under more strict conditions.

## 1. Introduction

Humans have used extracts derived from medicinal plants in folk medicine systems for thousands of years [1,2] and these traditions continue to the present day. According to the World Health Organization (WHO), ”a medicinal plant is defined as any plant which, in one or more of its organs, contains substances that can be used for therapeutic purposes, or which are precursors for chemo-pharmaceutical semi-synthesis” [3]. Many licensed drugs were originally derived wholly or partially from extracts of medicinal plants that had long histories of folk usage [4].

In 1995, the global market for medicinal herbs was approximately USD 17 billion, but a recent market analysis suggests that by 2023 this will increase to approximately USD 111 billion (compound annual growth rate of 7% to 8%). Market drivers for this increased demand in developed countries include the expense of insurance-based medical care, a rising desire of people in industrialized nations to take charge of their own health, and an increasing elderly population [5].

Some well-known systems of using herbal medicine are Ayurveda from India and Traditional Chinese Medicine (TCM), although the latter includes a variety of products of animal and fungal origin. Therefore, herbal products are beyond the scope of this review and are not systematically covered herein. The use of herbal or folk medicine has always been widespread in less developed countries, mainly because of a lack of modern healthcare. Often it is the primary, if not the only, means of treating the sick in 80% of Asian and African countries [6,7]. In developed countries, herbal medicines are also consumed. The United States Food and Drug Administration (US-FDA) estimated a decade ago that more than 20,000 different plant species were used in herbal remedies each year, and that one out of five US citizens used herbal medicines regularly [4]. In the European Union (EU), France, and Germany are the main markets for herbal remedies [5].

One of the most serious issues surrounding the safety of medicinal herbs concerns regulating the level of mycotoxins found in herbal preparations. Mycotoxins are a group of around 400 toxic secondary metabolites produced by fungi such as *Aspergillus, Penicillium, Fusarium, Claviceps,* and *Alternaria*. A detailed treatment of the effects of these fungal toxins is beyond the scope of this review; for an exhaustive survey see Selwet [8] and Zain [9]. Mycotoxins have previously been defined as ”fungal metabolites that when ingested, inhaled or absorbed through the skin, cause illness or human and animal death” [10,11,12]. Collectively mycotoxins can cause carcinogenic and other manifold toxic effects [13,14].

The mycotoxins most damaging to the health of humans and domestic animals are aflatoxins (AF), ochratoxin A (OTA), fumonisins (FB), zearalenone (ZEN), and deoxynivalenol (DON) [15,16,17,18]. The toxic properties, their occurrence, and their relevance in foods and feeds have been the subject of numerous comprehensive reviews, including the JECFA (Joint FAO/WHO Expert Committee on Food Additives), IARC (International Agency for Research on Cancer), EFSA (European Food Safety Authority), and other agencies [15,16,17,18,19,20,21]. Issues of analysis with regard to mycotoxins in herbal medicines have been reviewed recently by Zhang et al. [22].

## 2. Regulations Concerning Production and Distribution of Medicinal Herbs

Medical pharmacopeias, such as the European Pharmacopeia, are part of the strict legal frameworks used to regulate licensed drugs. Any compound included in a pharmacopeia is subject to very stringent pre- and post-market checks before it can be offered for sale. The product should meet the quality standards in the respective pharmacopoeias of the Member State or the European Pharmacopoeia. Bibliographic evidence should confirm the existence of a product in medicine for at least 30 years, including at least 15 years in the European community. [23]. Most herbal products are treated as ”food supplement”, ”herbal supplement”, or ”traditional medicine” as part of a ”fast track” regulatory system, whereby plants that are deemed (a) non-toxic and (b) have a written tradition of medicinal use are subject to less scrutiny. This approach is used particularly in the US, EU, Canada, and Australia [24,25].

International trade of herbal plants is subject to compliance with international treaties including the “Convention on International Trade of Endangered Species of Wild Fauna and Flora” (CITES). The CITES treaties deal with the control of international trade in certain species that are threatened with extinction. Herbal products are subject to various classifications, including complimentary medicines, natural health products, prescription medicines, over the counter medicines, supplements, traditional herbal medicines, etc. [26]. While drugs are tightly regulated, the scope of control over supplements is relatively low. In order to harmonze the classification of traditional preparations in countries where there is no established system of regulation in the field of traditional medicine, the WHO published a series of Traditional WHO Medicine Strategy papers for 2002 to 2005 and for 2014 to 2023. The strategy for 2002 to 2005 includes shaping government policy; ensuring safety, efficiency and quality; improving access; and promoting the proper use of traditional and complementary medicines [27]. The current strategy for 2014 to 2023 has two key objectives, first, to support member states in using the potential contribution of traditional and complementary medicines in health, well-being, and healthcare focused on people, and second, promoting safe and effective use of these medicines through regulation of products, practices, and practitioners [28].

The Scientific Panel for the European Food Standards Agency (EFSA) developed a document which provided an evidence-based framework against which the safety of botanical preparations could be judged [19]. This framework was based on the earlier recommendations of Schilter et al. [29]. Then, the EFSA further developed a two-level tiered system for the licensing of botanical preparations [30]. The European Medicine Agency (EMA) has laid down different methods of registering herbal medicinal products. The first is a full marketing authorization by submission of a dossier, which provides the information under directive 2001/83/EC. The second method is used for traditional herbal medicinal products, whereby a simplified procedure under regulation 2004/24/EC is followed [31,32].

The United States Food and Drug Administration has classified botanical products as drug, food or a dietary supplement on the basis of claims or end uses thereof. As per the FDA, a drug must be marketed under an approved New Drug Application (NDA), while dietary supplements are regulated under the Dietary Supplement Health and Education Act of 1994 [23]. Table 1 shows a compilation of the European Union, US-FDA, and European Pharmacopeia regulations with regard to mycotoxins in various foodstuffs.

Other countries have set similar regulations, although the maximum tolerated levels can vary. For example, the maximum limit for aflatoxin in India appears to be much higher (30 µg/kg) than in other countries [37,38]. In China, 5 to 20 µg/kg is the maximum limit depending upon the classification of the medicinal herb under consideration [33,36,39]. Mycotoxin contamination is particularly serious in India as many of the companies with the largest worldwide market share of medicinal herb sales are located there [15]. Indeed, there have been so many instances of problems with food imports from India containing high levels of mycotoxins that current EU regulations require 50% of all shipments of *Capsicum* (chili powders and peppers), *Zingiber officinale* (ginger), and *Curcuma longa* (turmeric) be tested for mycotoxins (EC 669/2009).

## 3. Data on Contamination of Selected Medicinal Plants by Mycotoxins

Mycotoxin contamination of selected medicinal plants belonging to the European Pharmacopoeia are now discussed, specifically for liquorice, chamomile, mint, ginseng, milk thistle, and ginger [36].

### 3.1. Glycyrrhiza Glabra (Liquorice)

Liquorice is a sweet extract from the root of *Glycyrrhiza glabra* L. which is frequently used in confectioneries. The main flavoring ingredient, glycyrrhizin, has various pharmaceutical properties. For example, it acts as an expectorant, has anti-inflammatory properties, and increases blood pressure. Excessive consumption of liquorice can also have adverse health effects. Despite the known health risks, liquorice is part of both TCM and Ayurveda [39]. Although liquorice itself is not susceptible to pests, the root can be infected by toxigenic fungi postharvest. This can be worsened by damages caused by aphid infestations, to which the plant seems to be quite susceptible. Several studies report relatively high levels of AFs and OTA in liquorice roots and in commercial liquorice products such as confectionary items. Specifically, the levels of OTA exceeded 100 µg/kg in some highly contaminated root samples, and thereby also exceeded the European Union maximum level in these products. In finished products, levels were lower but still at a notable level. In this context, it is important to consider that liquorice is frequently used as a sweetener in tea beverages for infants of less than one year of age [40]. The overall frequency and levels of AFs were considerably lower, and reports on the occurrence of other mycotoxins are largely anecdotal. The data obtained for mycotoxins in liquorice roots were mostly reported for food products, and analyses of liquorice products as herbal medicines are scarce. Tan et al. [41] in a survey of 138 TCMs from the Chinese market for Fusarium toxins T-2 and HT-2 found that only one sample of barley malt (*Hordeum vulgare*) was positive for T-2, while all three samples of liquorice were negative (Table 2). However, other research conducted for fumonisins showed their lack in products [42,43] or in the case of Roy et al.’s work [44], a low level.

### 3.2. Matricaria Chamomilla (Chamomile)

*Matricaria chamomilla* L. is the most popular source of the herbal product chamomile, although other species are also used as ”chamomile”. The raw material is used internally in the treatment of gastrointestinal symptoms such as spastic stomach cramps, flatulence, belching, and inflammation of the bile duct. Externally, it is recommended to reduce local inflammation of the skin and mucous membranes, through rinsing the mouth and throat, inhalation during irritation of the upper respiratory tract, baths, rinses, and ointments for inflammation around the anus and genital organs [52]. Harvesting is carried out between the second week of March and the third week of April, when weather conditions create a favorable environment for the development of pathogens. The dried chamomile is exposed to microbiological deterioration by fungal factors in a very short time. Thus, in the first stage, molds of the most xerophilic species, *Aspergillus* and *Penicillium*, are formed. By releasing increasing amounts of moisture, these molds provide favorable conditions for more demanding organisms such as *Fusarium* and *Rhizopus*. This increases the chances that the stored product is contaminated with mycotoxins that pose a health risk [52]. Tosun and Arslan [49] analyzed chamomile for the incidence of AF and FBs and reported relatively high levels of AFs in 9 of 10 samples, with a range of 3.4 to 38.9 µg/kg. Santos et al. [21] found the highest concentrations of AF in the all samples (35.8 to 161.0 µg/kg) in two samples of flower chamomile. Other reports showed that the dried raw chamomile products were on the lower level of mycotoxin contamination (Table 3).

### 3.3. Mentha sp. (Mint)

*Mentha sp.* is a genus of plants from the family *Lamiaceae*. Mint is a group of perennial aromatic plants that grow widely across Europe, Africa, Australia, and North America. For centuries, mint has been used as medicinal plant for gastrointestinal and respiratory ailments, bad breath, and dandruff and as a carminative, antispasmodic, diuretic, and sedative agent [54]. It grows in various habitats, most often wet, but also on dry steppes. They are grown mainly for the smell and taste of the leaves. For example, leaves of peppermint (*Mentha piperita*,) are used to obtain menthol oil which is used in the perfumery, pharmaceutical, and food industries (including the manufacture of sweets and cakes), and tobacco. Leaves of green mint (*Mentha spicata*) are used directly as a spice and also as a flavorant in chewing gum and toothpastes [55]. Research on mycotoxins in final mint products, mainly in the food industry, showed a low level of mycotoxicological contamination. However, Tosun and Arslan [49] showed that in two samples out of five tested, the level of AF exceeded the European Union acceptable norm (Table 4).

### 3.4. Panax Ginseng (Ginseng)

*Panax ginseng* C.A.Mey belongs to the *Araliaceae* family and is found throughout East Asia and Russia [62]. It grows wild in remote forests of Manchuria and North Korea but has become over-harvested in other parts of Asia. It is cultivated in Korea, China, and Japan for export and is widely used as a medicinal herb [63]. Although ginseng has been used by Asian cultures for thousands of years for conditions such as fatigue, mental stress, blood sugar regulation, improving libido, and supporting longevity, modern clinical studies have focused on the use of *P. ginseng* in blood sugar regulation, fatigue, and immunomodulation in human health and disease [64]. The plants grow in fertile, permeable humus soils with a slightly acidic pH. Because they do not tolerate direct sunlight, plants must be artificially shaded. Thus, ginseng cultivation is very demanding and, in addition, the growing conditions increase the risk of contamination with molds that produce mycotoxins. Therefore, the plant has been studied for mycotoxicological contamination. The results showed that sampled ginseng roots had significant levels of AFs which exceeded acceptable standards [38,65] (Table 5).

### 3.5. Silybum Marianum L. (Milk Thistle)

*Silybum marianum* L. is commonly known as milk thistle, Marian thistle, Saint Mary’s thistle, Mediterranean milk thistle, variegated thistle and Scotch thistle. This herb is an annual or biennial plant of the Asteraceae family and displays red to purple flowers and shiny pale green leaves with white veins. Originally, a native of Southern Europe through to Asia, it is now found throughout the world. Medicinally, the fruit is harvested to produce the antioxidant silymarin, since the levels of this compound make up to 2% to 3% of the fruit by weight. Silymarin is used both prophylactically (e.g., it is administered to people working with chemical vapors and ionizing radiation), as well as to promote healing in cirrhosis, hepatic steatosis, cholangitis, cholelithiasis, jaundice, and during cancer treatment. [71]. Like other herbs, this medicinal herb has been examined for mycotoxin contamination. Reports of research carried out in various centers have shown some cases of significant levels of contamination by mycotoxins, in particular AF, DON, and T-2 and HT-2, exceeding the allowed maximum limits. The results were primarily found in dietary supplements and products aimed at pregnant women. Therefore, reports that showed the presence of mycotoxins should be confirmed in order to reduce the health risks for consumers (Table 6).

### 3.6. Zingiber Officinale (Ginger)

Ginger (*Zingiber officinale Roscoe*) is a perennial root plant cultivated in tropical and subtropical climates. Due to the presence of bioactive components, the rhizomes are used primarily as a spice, but also for medicinal purposes. The increase in demand for ginger in the USA and Europe as a botanical dietary supplement is for treatment of chronic inflammation. Recent studies have demonstrated antioxidant and anti-emetic effects [75]. Products are delivered to Europe mainly from Nigeria and China, where, due to climatic conditions, they are exposed to contamination with secondary mold metabolites, usually at every stage of production, from harvesting to distribution itself [76]. As ginger is especially exposed to aflatoxins and ochratoxin A, the European Commission has set maximum levels of 5 mg/kg for AFB_1_, 10 mg/kg for all AFs (sum of AFB_1_, AFB_2_, AFG_1_, and AFG_2_) and 15 mg/kg for OTA [33,77]. As with other herbs, ginger has been studied for mycotoxin status. The results showed that in the tested samples there were samples which were contaminated with mycotoxins, but their levels did not exceed the acceptable norm, with the exception of two trials where the concentration of AFs exceeded acceptable standards [49] (Table 7).

## 4. Conclusions

The occurrence of mycotoxins in herbal products is still quite limited. Considering the rapidly increasing market, it is recommended that more effort should be put into the control of mycotoxins and other contaminants in plant products [81,82]. The quality and safety of herbal products are closely related to compliance with the principles of good agricultural practice at every stage of production. Plants classified as medical material should be carefully stored and checked for the presence of mycotoxins in order to protect the consumers who use these products to improve their health. The dose of herbal intake depends on the health of the consumer, and thus the level of mycotoxin contamination varies, and therefore the level of mycotoxins should be considered individually for each type of medicinal plant [83]. One of the challenges of international trade is the lack of common guidelines and a monitoring body to enforce the regulation of mycotoxins in herbal plants and for their products, especially for dietary supplements [84]. For many of these foods, the average consumption is low, therefore, the quantitative daily intake of mycotoxins via herbs, is possibly very low in many cases. However, the consumers’ trust in such products requires the absence of avoidable levels of toxins. The complex composition of herbal products affects their quality. Poor-quality raw materials can affect the final product. [85] The agricultural production of herbs under suboptimal circumstances may be an important contributing factor to toxin levels. Although aspects of risk-benefit assessments are applicable here, mycotoxins are a consequence of fungal growth, and therefore also an indicator of hygienic deficits during production and storage. In recent years, extensive research has been conducted with regard to biological, chemical, and physical strategies for the degradation and decontamination of mycotoxins [86]. An example of mycotoxin removal is cold atmospheric pressure plasma (CAPP), which is appropriate for sensitive biological stuffs [87]. Therefore, the option for solving the problem of mycotoxins is also implementation of the Good Agricultural and Collection Practices (GACP) and the Good Manufacturing Practices (GMP) [85]. Their presence in herbal products should be used as a stimulus to improve food quality and safety, by conducting further studies on the presence of mycotoxins in such products.

## Figures and Tables

**Table 1:** Regulations for mycotoxins in food.

Mycotoxins	Food Category	European Union, Regulation 1881/2006 [µg/kg] [33,34,35]	USA, FDA action level [µg/kg]	European Pharmacopeia 2.8.18. [µg/kg] [36]
Aflatoxin B_1_ (Sum of B_1_, B_2_, G_1_ and G_2_)	Herbal drugs	-	-	<2 (4)
	Ginger	5 (10)	-	-
	Dried figs	6 (10)	-	-
	Cereal-based foodstuffs	2 (4)	20	-
	Processed cereal-based foods and baby foods for infants and young children	0,10	-	-
Deoxynivalenol	Cereal-based foodstuffs	200–750	1000	-
Ochratoxin A	Liquorice root, ingredient for herbal infusion Liquorice extracts for use in food	20 80	-	20 80
	Ginger	15	-	-
	Cereal-based foodstuffs	3–5	-	-
	Processed cereal-based foods and baby foods for infants and young children	0,5	-	
Patulin	Foodstuffs	10–50	50	-
Zearalenone	Cereal-based foodstuffs	20–100	-	-

**Table 2:** Analysis of the literature available on mycotoxin contamination in liquorice and liquorice products.

Mycotoxins	Sample Type	Number of Samples	Percentage of Positive Results (%)	Range of Positive Results (µg/kg)	Percentage of “Critical”* Positive Samples (%)	Reference
AF	Liquorice root	1	100	0.59	0	[45]
OTA	Liquorice root	19	47	n.d.–216.5	22	[40]
	Liquorice sweet	19	95	n.d.–3.0	0	
AF	Liquorice root	10	40	n.d.–0.17	0	[44]
OTA		10	20	n.d.–0.05	0	
FB	Liquorice	1	0	n.d.	-	[43]
OTA	Dried liquorice roots	15	100	1.4–252.8	7	[46]
	Fresh liquorice root	8	100	3.3–14.7	0	
	Liquorice sweets	4	100	0.5–8.2	0	
	Liquid liquorice	2	100	14.6–17.3	0	
	Liquorice block	1	100	39.5	100	
FB	Liquorice	1	100	647	100	[21]
OTA	Dried Liquorice Liquorice confectionery	28 54	100	26.3–990.1	100	[47]
			61	<LOD–8.3	0	
AF			18	<LOD–2.4	20	
			15	<LOD–7.7	12,5	
OTA	Liquorice	1	100	0,2	0	[48]
T-2, HT-2	138 TCM samples including 3 samples of liquorice roots	3	0	n.d.	-	[49]
AF	Liquorice root	21	14	<LOQ–26.11	33	[50]
OTA			5	<LOQ–18.73	0	
AF	Liquorice root	4	50	n.d.–9.34	0	[51]
OTA			75	n.d.–13.1		
CIT DON FB_1_ OTA ZEN	Liquorice	31	6	6.75–20.44	50	[42]
			3	<LOQ–11.08	0	
			6	<LOQ–39.34	0	
			3	<LOQ–3.93	0	
			13	3.37–8.75	0	

* Critical-under the UE regulations limits of mycotoxins; AF-Aflatoxin, OTA-Ochratoxin A, FB-Fumonisin, ZEN-Zearalenone, DON-Deoxynivalenol, CIT-Citrinin.

**Table 3:** Analysis of the literature available on mycotoxin contamination in chamomile.

Mycotoxins	Sample Type	Number of Samples	Percentage of Positive Results (%)	Range of Positive Results (µg/kg)	Percentage of “Critical”* Positive Samples (%)	Reference
FB	Chamomile	18	45	20–70	0	[53]
FB	Chamomile	1	0	-	0	[43]
OTA,	Chamomile flower	2	100	0.8–1.0	0	[21]
FBs,			50	<LOD–90.0	0	
AF,			100	35.8–161.0	100	
ZEN,			100	7.3–12.5	0	
T-2,			100	3.5–8.3	0	
DON,			100	123.4–191.5	0	
CIT			100	31.7–49.3	100	
AF	Chamomile	10	100	3.4–38.9	90	[49]
FB			0	-	-	

* Critical-under the UE regulations limits of mycotoxins; AF-Aflatoxin, OTA-Ochratoxin A, FB-Fumonisin, ZEN-Zearalenone, DON-Deoxynivalenol, CIT-Citrinin.

**Table 4:** Analysis of the literature available on mycotoxin contamination in mint.

Mycotoxins	Sample Type	Number of Samples	Percentage of Positive Results (%)	Range of Positive Results (µg/kg)	Percentage of “Critical”* Positive Samples (%)	Reference
AF	Mint	2	0	-	-	[56]
OTA			0	-	-	
ZEN			0	-	-	
FB	Mint	1	0	-	-	[57]
OTA			0	-	-	
AF	Mint	5	0	-	-	[58]
AF	Mint	6	0	-	-	[59]
AF	Mint	10	0	-	-	[60]
OTA,	Mint	2	100	1–1.4	0	[21]
FBs,			0	<LOD	0	
AF,			100	16.6–29.7	100	
ZEN,			100	2.1–9.3	0	
T-2,			100	3.9–4.9	0	
DON,			100	46.9–91.1	0	
CIT			100	41.0–43.3	100	
FB_1_	Mint	2	50	n.d.–0.160	0	[43]
FB_2_			0	n.d.	-	
AF	Mint	5	80	4.2–26.7	50	[49]
AF	Mint	31	29	0.3–2.19	11	[61]
OTA			45	0.38–12.32	0	
HT-2			0	-	-	
T-2			3	3.81	0	
FB			61	<LOQ–102.32	0	
ZEN			84	0.11–44.74	0	

* Critical-under the UE regulations limits of mycotoxins; AF-Aflatoxin, OTA-Ochratoxin A, FB-Fumonisin, ZEN-Zearalenone, DON-Deoxynivalenol, CIT-Citrinin.

**Table 5:** Analysis of the literature available on mycotoxin contamination in ginseng.

Mycotoxins	Sample Type	Number of Samples	Percentage of Positive Results (%)	Range of Positive Results (µg/kg)	Percentage of “Critical”* Positive Samples (%)	Reference
AF	Ginseng root	12	17	15.1–15.2	100	[66]
AF	Ginseng	10	30	<0.1	0	[67]
OTA			40	0.4–1.8	0	
AF	Ginseng	76	0	-	-	[64]
AF	Ginseng	2	0	n.d.	-	[68]
OTA			100	3.2	0	
OTA	Ginseng	13	0	-	-	[69]
FB			0	-	-	
AF	Ginseng	10	0	0	-	[65]
OTA			0	0	-	
HT-2			0	0	-	
T-2			0	0	-	
ZEN			0	0	-	
CIT			0	0	-	
FB			10	<10	0	
AFB1	Ginseng root	7	29	48.8–143	100	[38]
AFB2				18.6–355	100	
OTA				-	-	
AFB1	Notoginseng radix et rhizoma	3	100	1.29–2.1	0	[70]
AFB2			0	-	-	
AFG1			0	-	-	
AFG2			0	-	-	
OTA			0	-	-	

* Critical-under the UE regulations limits of mycotoxins; AF-Aflatoxin, OTA-Ochratoxin A, FB-Fumonisin, ZEN-Zearalenone, CIT-Citrinin.

**Table 6:** Analysis of the literature available on mycotoxin contamination in milk thistle.

Mycotoxins	Sample Type	Number of Samples	Percentage of Positive Results (%)	Range of Positive Results (µg/kg)	Percentage of “Critical”* Positive Samples (%)	Reference
OTA,	Milk thistle	2	0	<LOD,	-	[21]
FBs,			50	<LOD–236.7	100	
AF,			100	10.9–11.5	100	
ZEN,			100	1.6–3.5	0	
T-2,			100	17.5–35.6	50	
DON,			0	<LOD	-	
CIT			0	<LOD	-	
AF	Milk thistle	83	16	0.04–2.0	0	[72]
T-2	Milk thistle	2	100	363.0–453.9	100	[73]
HT-2		2	100	826.9–943.7	100	
ZEN		1	100	<LOD	0	
DON	Milk thistle	32	3	2890	100	[74]

* Critical-under the UE regulations limits of mycotoxins; AF-Aflatoxin, OTA-Ochratoxin A, FB-Fumonisin, ZEN-Zearalenone, DON-Deoxynivalenol, CIT-Citrinin.

**Table 7:** Analysis of the literature available on mycotoxin contamination in ginger.

Mycotoxins	Sample Type	Number of Samples	Percentage of Positive Results (%)	Range of Positive Results (µg/kg)	Percentage of “Critical”* Positive Samples (%)	Reference
AF	Ginger	26	50	0.12–0.85	0	[78]
OTA				0.01–0.09	0	
CIT				0.00–0.02	0	
ZEN	Ginger	27	7	13.44–14.51	0	[79]
DON			15	4.85–10.35	0	
AF	Ginger	4	75	3.8–23.1	67	[49]
AFB_1_	Ginger	30	17	0.13–1.38	0	[80]
OTA				0.31–5.17	0	
AF OTA	Ginger rainy	31	81	0.11–9.52	0	[76]
			77	0.20–9.90	0	
	Ginger dry	89	46	0.20–3.57	0	
			37	0.17–12.02	0	

* Critical-under the UE regulations limits of mycotoxins; AF-Aflatoxin, OTA-Ochratoxin A, ZEN-Zearalenone, DON-Deoxynivalenol, CIT-Citrinin.
